# Effects of therapeutic hypothermia on brain function in a refractory cardiac arrest model treated with extracorporeal cardiopulmonary resuscitation

**DOI:** 10.1186/s40635-025-00841-w

**Published:** 2025-12-15

**Authors:** Anthony Moreau, Fuhong Su, Lorenzo Ferlini, Nicolas Gaspard, Francesca Pischiutta, Elisa Zanier, Jacques Creteur, Filippo Annoni, Fabio Silvio Taccone

**Affiliations:** 1 Department of Intensive Care, Hôpital Universitaire de Bruxelles (HUB), Université Libre de Bruxelles (ULB), Route de Lennik, 808, 1070 Brussels, Belgium; 2 Laboratoire Expérimental des Soins Intensifs, Université Libre de Bruxelles (ULB), Brussels, Belgium; 3 Department of Neurology, Hôpital Universitaire de Bruxelles (HUB), Université Libre de Bruxelles (ULB), Brussels, Belgium; 4 Neurology Department, School of Medicine, Yale University, New Haven, CT USA; 5 Laboratory of Traumatic Brain Injury and Neuroprotection, Department of Acute Brain Injury, Istituto Di Ricerche Farmacologiche Mario Negri IRCCS, Via Mario Negri 2, 20156 Milan, Italy

**Keywords:** Hypoxic-ischemic brain injury, Multimodal neuromonitoring, Refractory cardiac arrest, ECPR, Systemic hypothermia

## Abstract

**Background:**

Cardiac arrest (CA) remains a leading cause of mortality and long-term neurological disability. In cases of refractory CA, extracorporeal cardiopulmonary resuscitation (ECPR) may be implemented as a salvage therapy to mitigate hypoxic-ischemic brain injury and improve outcomes. However, the optimal target temperature in the specific context of ECPR remains uncertain. The objective of this study was to evaluate the impact of hypothermia on brain function using a controlled experimental model of ECPR.

**Methods:**

Twelve pigs were subjected to 5 min of untreated ventricular fibrillation, followed by 25 min of conventional cardiopulmonary resuscitation (CPR). At 30 min, veno-arterial extracorporeal membrane oxygenation support was initiated, and defibrillation attempts were performed until the achievement of return of spontaneous circulation (ROSC). Following ROSC, animals were randomly assigned to one of two groups: hypothermia (HT), targeting a core body temperature of 33–34 °C, or controlled normothermia (NT), targeting 37–38 °C. All animals underwent continuous multimodal neurological and cardiovascular monitoring. Blood samples were collected at predefined time points to assess circulating biomarkers of organ injury. The primary outcome was the change of brain tissue oxygen tension (PbtO_2_) over time. Other neurological and hemodynamical parameters were treated as secondary analyses. At 12 h post-ROSC, animals were euthanized via intracardiac injection of potassium chloride. Brain tissues were immediately harvested and appropriately stored for molecular analyses.

**Results:**

A total of 12 pigs were included in the study, with six animals allocated to each group. Baseline characteristics were comparable between the groups and ROSC was achieved in all animals. Throughout the experiment, PbtO₂ gradually declined and intracranial pressure (ICP) increased in both groups; however, no significant differences were observed between groups. Similarly, there were no significant differences in cerebral metabolites, cortical activity, or gene expression in either frontal or parietal brain tissues. Notably, neurofilament light chain (NfL) concentrations were significantly lower at the end of the observation period in the HT group compared to NT (*p* = 0.04), while neuron-specific enolase (NSE) and glial fibrillary acidic protein (GFAP) levels did not differ significantly between the two groups.

**Conclusions:**

HT did not improve cerebral perfusion or metabolic parameters in this refractory cardiac arrest ECPR model; the early decrease in NfL levels requires cautious interpretation and further investigation.

**Supplementary Information:**

The online version contains supplementary material available at 10.1186/s40635-025-00841-w.

## Background

Cardiac arrest (CA) imposes a significant burden on public health due to its high incidence and substantial number of deaths throughout the world [1]. Among patients resuscitated from CA, cardiovascular failure and hypoxic-ischemic brain injuries (HIBI) are the leading causes of unfavorable outcomes and deaths [2]. In order to improve the quality of the cardiopulmonary resuscitation (CPR), many efforts have been proposed over the last years, including the promotion of bystander CPR and early defibrillation [3]. However, even in some patients with witnessed CA and immediate CPR, conventional CPR might still be ineffective to achieve the return of spontaneous circulation (ROSC) and these patients might benefit from an extra-corporeal cardiopulmonary resuscitation (ECPR). Even though this intervention is still debated, ECPR may improve survival and neurological outcomes in selected patients’ populations [4–6].

In patients resuscitated from CA, therapeutic hypothermia (HT) has always generated considerable interest since its potential protective effects not only on brain, but also on other organs [7]. Besides diminishing the systemic and cerebral metabolism and consequent reduction in glucose and oxygen consumption, HT can also decrease carbon dioxide production, which would decrease the risk of intracranial hypertension [7, 8]. By inhibiting the caspase activation [9], HT would also prevent mitochondrial dysfunction and interrupt the apoptotic pathway. Furthermore, HT can attenuate the neuroexcitatory cascade, which occurs after cerebral ischemia–reperfusion [10], reduce the local neutrophil and macrophage activation [7, 11], and limit the production of free radicals [12].

Targeted temperature management (TTM), currently recommended by the European Resuscitation Council (ERC) and the European Society of Intensive Care Medicine (ESICM) for comatose patients after CA [13], aims to maintain body temperature below 37.7 °C. However, due to a prolonged resuscitation time and rapid reperfusion in ECPR patients, cerebral ischemia–reperfusion injuries could be more severe [14] and therefore, lower targeted temperatures might be beneficial in these patients. A recent narrative review [15] summarized the existing evidence on the use of HT and TTM in patients requiring extracorporeal membrane oxygenation (ECMO) therapy, particularly in ECPR patients. Although most of the selected studies were case reports or retrospective studies, the implementation of HT in ECPR patients appeared to be a feasible and promising intervention, supported by robust rationale and physiological background. In addition, compared to clinical studies evaluating HT in post-CA patients [16], ECPR allowed a significantly faster achievement of target temperature, thereby more closely replicating preclinical models in which rapid induction of HT has been associated with neuroprotective effects [17].

Beyond the theoretical neuroprotective mechanisms of HT, assessing its cerebral effects requires robust neuromonitoring. Multimodal neuromonitoring (MNM) has become essential to characterize secondary brain injury after CA, as it integrates complementary physiological signals to assess cerebral perfusion–metabolism coupling [18]. Among these modalities, brain tissue oxygenation (PbtO₂) represents a particularly informative parameter, offering a specific assessment of the balance between cerebral oxygen delivery and metabolic demand. PbtO₂ has been associated with neurological outcomes in post-cardiac arrest settings, supporting its relevance for evaluating early cerebral reperfusion and guiding targeted interventions [19].

In this study, we postulated that HT could reduce HIBI after refractory CA needing ECPR. We therefore assessed the effects of HT on brain perfusion, metabolism, cortical activity, and biomarkers of brain injury in a refractory CA animal model using ECPR.

## Methods

### Experimental setting

The Institutional Review Board for Animal Care of the Université libre de Bruxelles (ULB, Belgium) approved all experimental procedures (Ethical Committee approval number: 731 N), which were also in compliance with ARRIVE 2.0 (Animal Research: Reporting in Vivo Experiments) guidelines. Care and handling of the animals were in accord with National Institutes of Health guidelines (Institute of Laboratory Animal Resources). For all experiments, both sex swine of 6 months old and weighing 45–55 kg (*Sus scrofa domesticus)* were used.

### Animal preparation

A complete description of our experimental model developed in our laboratory was published previously [20]. Briefly, before the experiment, the animal fasted for 12 h with free access to water. After sedation in the cage with an intramuscular injection of midazolam (1 mg/kg) and ketamine (10 mg/kg) in the neck, the animal was placed on the operating table in a supine position. After establishing electrocardiogram monitoring and cannulating a marginal ear vein, rapid induction for orotracheal intubation using propofol (1.5 mg/kg), atropine (0.5 mg), morphine (3 µg/kg), and rocuronium (1.2 mg/kg) was initiated. Mechanical ventilation was started in controlled volume mode with standardized settings: tidal volume of 8 mL/kg, positive end-expiratory pressure of 5 cmH_2_O, fraction of inspired oxygen (FiO_2_) of 100%, and inspiratory to expiratory ratio of 1:2. Respiratory rate and FiO_2_ were then adjusted to provide a partial pressure of carbon dioxide (PaCO_2_) and a partial pressure of oxygen (PaO_2_) between 35 and 45 mmHg and 90 and 120 mmHg, respectively. A continuous venous infusion of rocuronium (1.5 mg/kg*h), morphine (0.2 mg/kg*h), and balanced crystalloids (300–500 ml/h, adapted to obtain a pulse pressure variation, PPV < 13%) was started, and a continuous inhaled sevoflurane was administered to achieve an expiratory percentage between 1.8 and 2.3%. With the aid of ultrasound, a three-lumen central venous catheter was placed in the right external jugular vein, an arterial catheter in the right radial artery, and an introducer for a pacing wire and a pulmonary artery catheter in the left external jugular vein. After an incision in the lower abdomen, a Foley catheter was surgically inserted into the bladder. Before the beginning of the instrumentation, 2 g of amoxicillin–clavulanate was administered to the animal.

### Neurosurgical procedure

A complete description of the implementation of MNM developed in our experimental laboratory was published previously [21]. Briefly, after turning the animal into sternal recumbency, a reverse mirrored F incision was executed on both sides, approximately 0.5 cm from the midline. Using an electric surgical drill, five burr holes were made in the skull. Through the caudal holes, two 5-contact intracranial stereo-electroencephalography (sEEG) wires (Microdeep 5 SEEG electrode, Dixi medical, France) were inserted. In the rostral holes, two 10-mm membrane length microdialysis catheters (one catheter CMA 20 and one catheter CMA 100, CMA microdialysis, Sweden) were placed. In the fifth hole, a catheter allowing the simultaneous collection of cerebral temperature (CT), intracranial pressure (ICP), and brain oxygen pressure (PbtO_2_; Neurovent PTO2, Raumedic AG, Germany) was introduced.

### Cannulation procedure and ECMO preparation

After turning the animal back into dorsal recumbency, one arterial (17Fr) and one venous (25Fr) cannula (Medtronic, Minneapolis, MN, USA) were placed under ultrasound control in the femoral artery and vein, respectively. Immediately after cannulation, a bolus of unfractionated heparin (100 UI/kg) was administered, followed by a continuous venous infusion (50 UI/kg*h). The cannulas remained clamped till the start of the ECPR and were flushed repeatedly with 50 ml of balanced crystalloids to avoid clot formation. At CPR initiation, the cannulas were then connected to the ECMO device. The ECMO circuit included a tubing set (EUROSETS, Medolla, MO, Italy), an oxygenator (EUROSETS, Medolla, MO, Italy), a centrifugal pump (affinity pump, Medtronic, Minneapolis, MN, USA), and a console (Transonic, Ithaca, NY, USA). The ECMO circuit was previously primed with balanced crystalloids.

### Cardiac arrest procedure

After a period of stabilization, ventricular fibrillation (VF) was induced by a catheter introduced into the right ventricle through the left external jugular vein and connected to a 9 V battery. Following CA, the animal was left untreated for a 5-min period (no-flow), and chest compressions were administered for 25 min (low-flow) using a mechanical chest compression device (Lucas III, Jolife AB/Stryker Lund, Sweden) at a rate of 100 compressions per minute, as previously reported [20]. Epinephrine was administered intravenously at intervals of 5, 10, 15, 20, and 25 min during mechanical compressions, each time at a dose of 30 µg/kg, followed by a flush of 10 ml of crystalloid solution. Thirty minutes after the induction of CA, mechanical compressions were halted, and ECMO was initiated (initial settings: blood flow 50 ml/kg*min, sweep gas flow 3 L/min, and FO_2_ 100%), and defibrillation attempts (4 J/kg biphasic electric shock) were performed as necessary. ROSC was defined as the establishment of an organized cardiac rhythm with a mean arterial pressure (MAP) exceeding 60 mmHg for a minimum of 10 min. Sweep gas flow was therefore adjusted to maintain a PaCO_2_ of 35–45 mmHg. A continuous infusion of norepinephrine was given to maintain a MAP between 65 and 70 mmHg. All animals received a fixed continuous infusion of balanced crystalloids (5–10 ml/kg*h); additional fluids were administered to keep PPV < 13%. Hyperglycemia after ROSC was left uncorrected. If supraventricular arrhythmia was observed, intravenous amiodarone (300 mg) was administered. Neurological, hemodynamic, and respiratory parameters were recorded hourly after ROSC. Twelve hours after ROSC, the animal was killed via an intracardiac potassium chloride injection.

### Group allocation and additional treatment

**Fig. 1:**
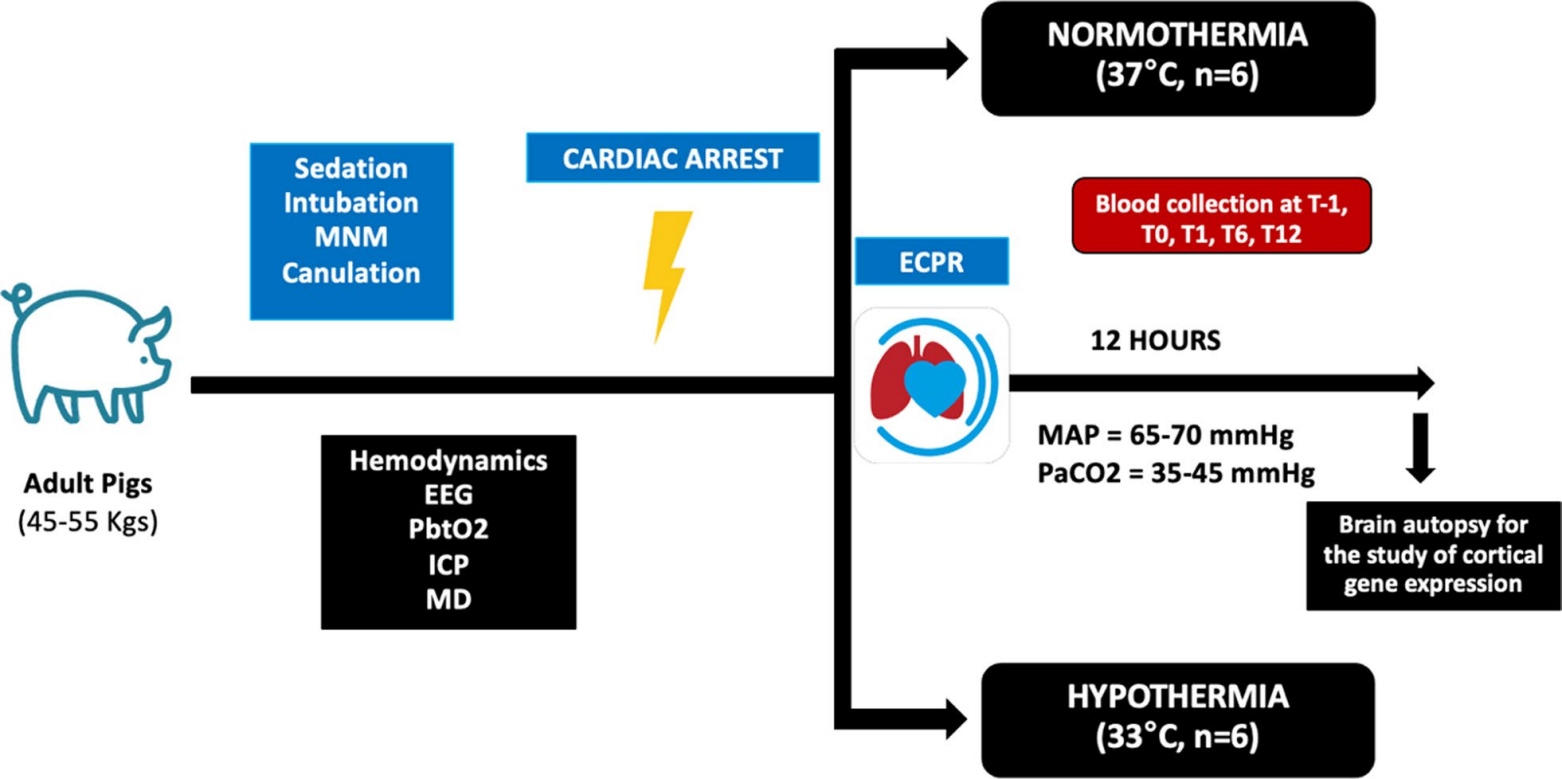
Protocol timeline. MNM: multimodal neuromonitoring; EEG: electroencephalography; PbO_2_: brain oxygen pressure; ICP: intracranial pressure; MD: microdialysis; ECPR: extracorporeal cardiopulmonary resuscitation; MAP: mean arterial pressure; PaCO_2_: partial pressure of carbon dioxide; T-1: baseline; T0: ROSC

On the day of the experiment, pigs were randomly assigned to the HT group (body temperature between 33 and 34 °C) and the NT group (body temperature between 37 and 38 °C) in a ratio of 1:1 (simple randomization). TTM was achieved using a heat exchanger connected to the ECMO circuit. Body temperature was controlled immediately after ROSC and for all the duration of the experiment. The timeline of the experiment is shown in Fig. 1.

### Blood, brain interstitial fluid and cerebral tissue sampling

Blood gas analyses were obtained at baseline (T-1), ROSC (T0), and every hour till the end of the experiment (from T1 to T12). Furthermore, arterial blood samples were collected at T-1, T0, T1, T6, and T12. Samples were then immediately centrifuged to obtain plasma and stored at – 80 °C. Microdialysis samples were obtained at baseline and hourly after ROSC.

For all microdialysis samples, glucose, lactate, pyruvate, glutamate, and glycerol were measured. Glutamate corresponds to excitotoxic release during ischemia, glycerol is a marker of cell membrane breakdown and structural injury, and the L/P ratio indicates shifts in cellular redox state and mitochondrial dysfunction [22, 23].

After killing the animal, the skull was opened, the dura mater was dissected using a scalpel, and cerebral tissues (frontal and parietal lobes) were harvested on each side. Tissues were either immediately frozen in liquid nitrogen and stored at − 80 °C or preserved in formaldehyde 4%.

### Outcomes

The primary outcome of our study is the change of PbtO_2_ over time. Other neurological and hemodynamical parameters are treated as secondary analyses.

### Data analysis

Physiological variables, including blood pressure (systolic, diastolic, mean), heart rate, and cardiac output, were recorded continuously and exported to a recording station (Notocord-Hem 4.4, Notocord, France). Neurological parameters (PbO_2_, ICP, and CT) were recorded using MPR2 logO DATALOGGER (Raumedic AG, Germany). Data were extracted at baseline, T0, and every hour till the end of the experiment. Blood gas analysis performed via a blood gas analyzer (Roche Cobas 123) allowed to measure pH, PaCO_2_, PaO_2_, lactate, and glucose concentrations. Arterial blood samples were used to measure hemoglobin, white blood cells, platelets, blood urea nitrogen, creatinine, aspartate aminotransferase (GOT), alanine aminotransferase (GPT), lactate dehydrogenase (LDH), total and direct bilirubin, troponin I, and creatine phosphokinase (SYNLAB Heppignies, Belgium).

Neurochemical markers (glucose, lactate, pyruvate, glycerol, glutamate) were measured using Clinical Cerebral Microdialysis Analyser (ISCUSflex microdialysis analyzer, Aurora Borealis Control B.V., The Netherlands). Samples from the second CMD were stored at –80 °C in case of additional analyses.

Plasma concentrations of glial fibrillary acid protein (GFAP) and neurofilament light chain protein (NfL) were measured using commercially available single molecule array assay kits (Quanterix, GFAP: #102,336; NfL: #103,400) on an SR-X analyzer according to the manufacturer’s instructions (Quanterix, Billerica, MA). A single batch of reagents was used for each analyte. Neuron-specific enolase (NSE) was measured using a commercially available ELISA kit following the manufacturer’s instructions (Cusabio, #CSB-E14065p). For gene expression analysis, total RNA was extracted from the cortex by using the Ribospin II extraction kit (Gene All) following the manufacturer’s protocol. Samples were reverse transcribed using TaqMan qPCR RT Master Mix (Applied Biosystems). Real-time reverse transcription PCR was performed, and the relative gene expression was determined by the ΔΔCt method with RPL27 as the housekeeping gene. Data are expressed as the log2-fold difference compared to the control group. The exploratory analysis included the following genes: microtubule-associated protein 2 (MAP2); GFAP; cluster of differentiation molecule 11ß (CD11ß); platelet and endothelial cell adhesion molecule 1 (PECAM1); caspase 3 and 8 (CASP3 and CASP8), and heme oxygenase 1 (HO-1), as previously described [24]. All analyses were performed blinded to treatment allocation.

All sEEG traces were acquired via recording station (Notocord-Hem 4.4, Notocord, France). During the CA procedure, the sEEG electrodes were disconnected to prevent electrical damage of the experimental tools from the shock and reconnected as soon as possible after ROSC. The recording lasted throughout the observation phase. The EEG signal with the best signal-to-noise ratio was visually selected for further analysis. All analyses were performed offline, using built-in and custom functions in MATLAB. We filtered the EEG signal between 1 and 15 Hz (4th-order Butterworth bandpass filter, *filtfilt* function in MATLAB) and then computed the Hilbert transform (*hilbert* function in MATLAB) of the filtered signal and extracted the amplitude, as previously reported [24]. We calculated the mean, kurtosis, skewness, and standard deviation of the amplitude using a sliding 1-min window with 50% overlap.

### Statistical analysis

The number of animals was restricted to six per group based on an *interim* statistical analysis. Given the absence of significant differences at this stage, further inclusions were considered unnecessary, and the sample size was therefore limited accordingly. The power calculation for the originally planned sample and the interim analysis plan were performed by the study investigators and subsequently confirmed by an external statistician, as detailed in the Supplementary Appendix. The interim analysis plan and predefined stopping rules were established before any data were collected. The interim analysis was unblinded, conducted by the study investigators according to the pre-specified statistical analysis plan. Statistical analysis was performed using Prism 9 (Version 9.1.2, San Diego, CA, USA). Continuous variables were expressed as mean with standard deviation (SD) or median with interquartile range and were compared using a t test or a Mann–Whitney test, as appropriate. Categorical variables were compared using Fisher’s exact test or a Chi-square test, as appropriate. For all longitudinal endpoints, a single pre-specified analytic approach was used. Specifically, multiple-group comparisons were performed using a mixed-effects model with Greenhouse–Geisser correction. The effects of time and group, as well as interactions between groups and time, were tested as fixed effects, and animals were introduced as random effects. If there were significant differences, the two-stage linear procedure of Benjamini, Krieger, and Yekutieli, with individual variances, was used to compare the means of these variables for the groups at each time point. A value of *p* < 0.05 was considered statistically significant.

## Results

### Baseline characteristics

Twelve pigs were included in the analysis (*n* = 6 in HT group, and *n* = 6 in NT group). All the animals achieved ROSC. Baseline characteristics of the study groups are shown in Table 1; there were no significant differences in body weight and in body temperature between the two groups. Hemodynamical parameters (e.g., cardiac output, mean arterial pressure, blood lactate levels) and neurological parameters (e.g., cerebral temperature, intracranial pressure, and brain oxygen pressure) were also comparable between the two groups at baseline.

**Table 1:** Baseline characteristics of the study groups

	NT group (*n* = 6)	HT group (*n* = 6)	*p* value
Body weight (kg)	53.5 ± 3.4	52.4 ± 4.5	0.55
Body temperature (°C)	36.5 ± 0.8	35.7 ± 0.7	0.06
Cardiac output (L/min)	6.5 ± 0.9	6.4 ± 0.7	0.83
Mean arterial pressure (mmHg)	73 ± 4	68 ± 9	0.30
Blood lactate (mmol/L)	1.2 ± 0.2	1.3 ± 0.4	0.56
Blood glucose (mg/dL)	98 ± 28	126 ± 25	0.08
Cerebral temperature (°C)	36.8 ± 0.5	35.7 ± 0.7	0.06
PbtO_2_ (mmHg)	51.3 ± 12.1	45 ± 10.8	0.46
ICP (mmHg)	8.7 ± 3.7	9.8 ± 2.9	0.56

Data are presented as count (%) or mean ± SD

NT: normothermia; HT: hypothermia; PbtO_2_: brain tissue oxygen pressure; ICP: intracranial pressure

*p* < 0.05 was considered as statistically significant

### Sedation, analgesia, and respiratory support parameters

After ROSC, no statistically significant difference was observed between the HT group and the NT group in terms of ventilation and ECMO sweep gas settings, sedative and analgesic doses over time, and depth of anesthesia (expressed as Sevoflurane MAC). All the results are provided in the Supplemental Appendix (Figure S1).

### Physiological and metabolic variables

**Fig. 2:**
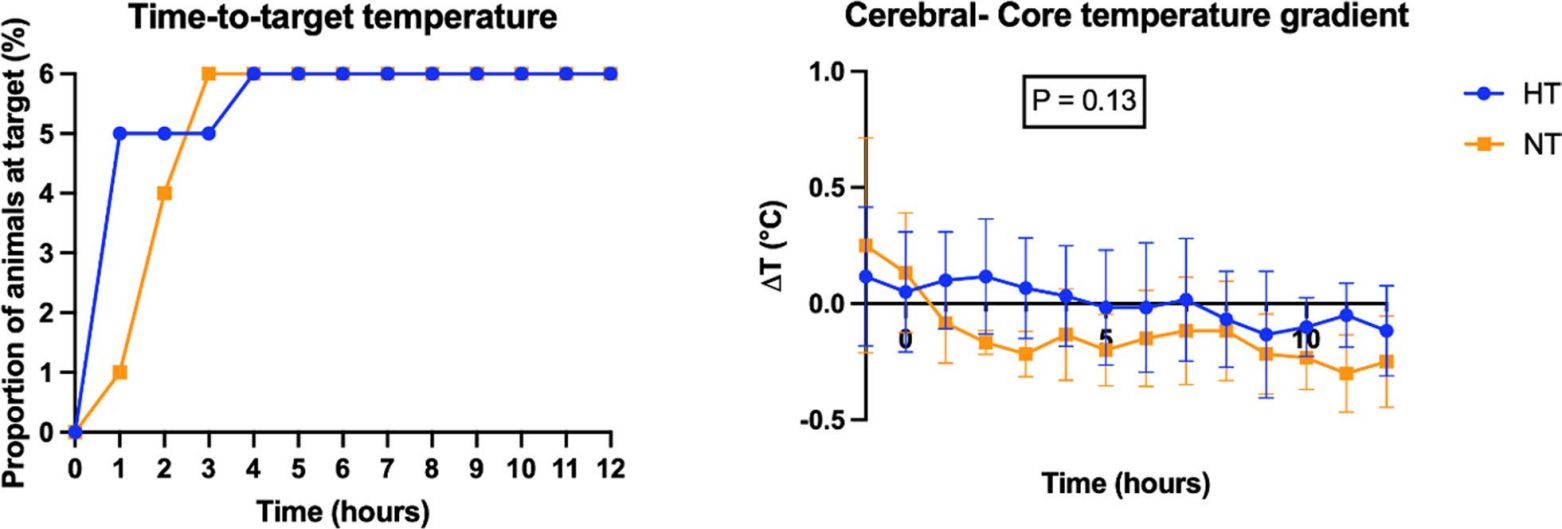
Time-to-target temperature and cerebral–core temperature gradient (ΔT) in HT and NT group

After ROSC, the body temperature was statistically significantly different between the two groups throughout the whole experiment (37.2 ± 0.6 °C in NT group; 33.6 ± 0.6 °C in HT group—Fig. 2) (*p* *group × time interaction* < 0.0001), whereas no statistically significant differences were observed between the HT group and the NT group in terms of pH, PaO_2_, and PaCO_2_. For the temperature variable that showed a statistically significant group × time interaction, the corresponding effect sizes and their 95% confidence intervals are presented in the Supplementary Appendix (Table S2).

The proportion of animals that reached the target temperature over time is shown in Fig. 2. Cooling was rapid and consistent in the HT group, with 5/6 animals (83%) reaching the target within 1 h and all animals (100%) by 3 h. In the NT group, cooling progressed more gradually: 1/6 animal (17%) reached the target at 1 h, 4/6 (67%) at 2 h, and all animals by 3 h. In the HT group, no overshoot or undershoot events were observed over 12 h (6 animals, hourly measurements). In the NT group, 3 of 6 animals (50%) exhibited transient overshoot episodes above the target range (2, 3, and 1 event, respectively; total = 6 events during 72 recorded hours), while the remaining 3 animals maintained temperature within target limits. The cerebral–core temperature difference (ΔT) is defined as the difference between the cerebral temperature and the core temperature. ΔT remained minimal and stable throughout the 12-h observation period in both groups. Mean ΔT values fluctuated around zero, indicating comparable brain and core cooling. In the linear mixed-effects model, a small effect of time was observed (*p* = 0.009), but neither the group effect (HT vs NT, *p* = 0.13) nor the group × time interaction (*p* = 0.22) reached significance (Fig. 2).

### Hemodynamical parameters

**Fig. 3:**
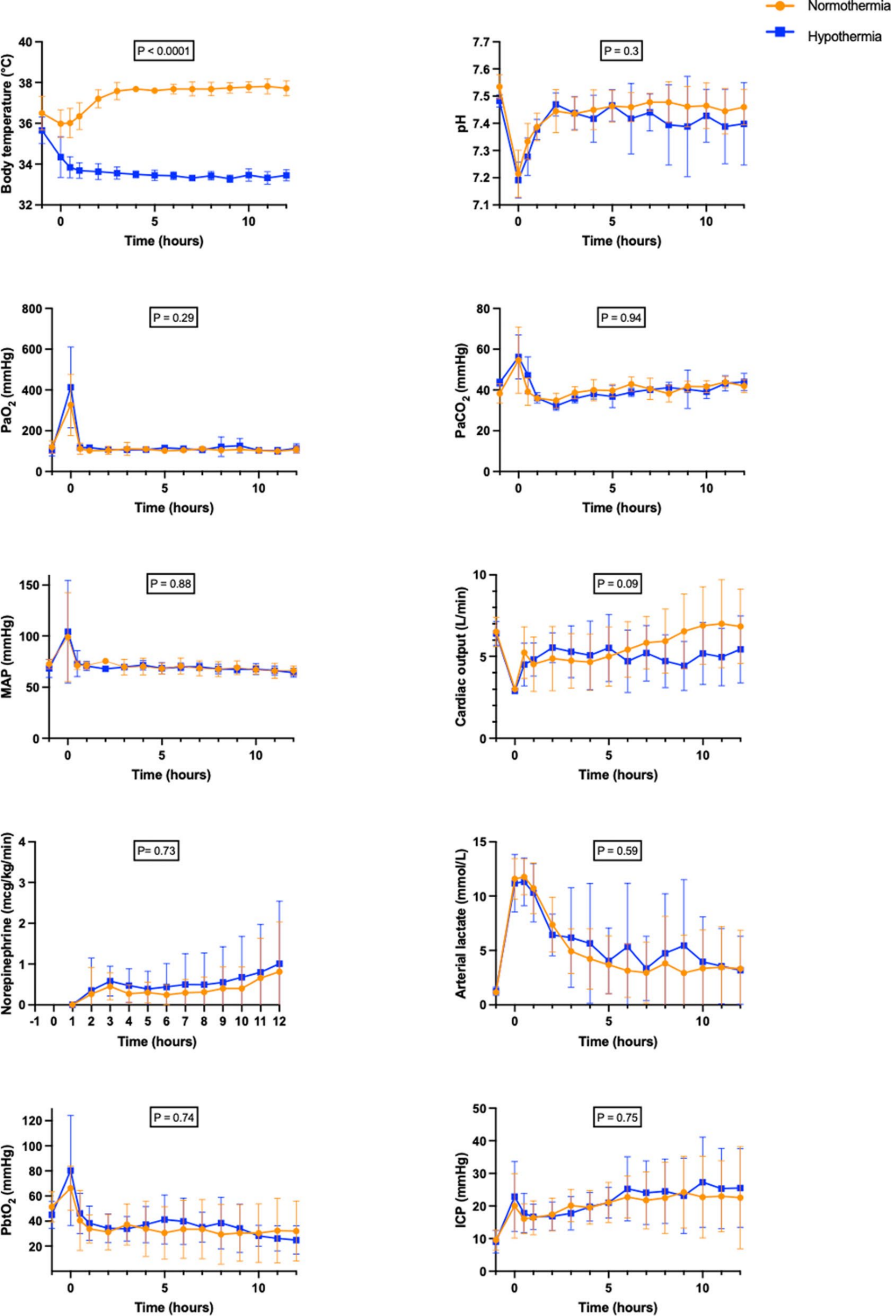
Time-course of physiological parameters, metabolic variables, hemodynamical parameters, PbO_2_ and ICP levels throughout the experiment. PaO_2_: partial pressure of oxygen; PaCO_2_: partial pressure of carbon dioxide; MAP: mean arterial pressure; PbtO_2_: brain tissue oxygen pressure; ICP: intracranial pressure. *p* *group x time interaction* < 0.05 was considered as statistically significant

After ROSC, no statistically significant difference was observed between the HT group and the NT group in terms of cardiac output, mean arterial pressure, norepinephrine dose, and lactate levels (Fig. 3). Fluid balance was also not significantly different between the HT group (8815 ± 1664 mL) and the NT group (9603 ± 758 mL; *p* = 0.93).

### Cerebral perfusion

After ROSC, no statistically significant difference was observed between the HT group and the NT group regarding PbtO_2_ and ICP values over time (Fig. 3). Mean ICP was 19.9 mmHg in the NT group and 21.1 mmHg in the HT group, corresponding to a non-significant mean difference of − 1.18 mmHg (95% CI − 9.17 to + 6.82). Mean PbtO₂ was 39.1 mmHg in the HT group and 36.4 mmHg in the NT group, corresponding to a non-significant mean difference of − 2.65 mmHg (95% CI − 19.9 to + 14.6).

### Cerebral metabolites

**Fig. 4:**
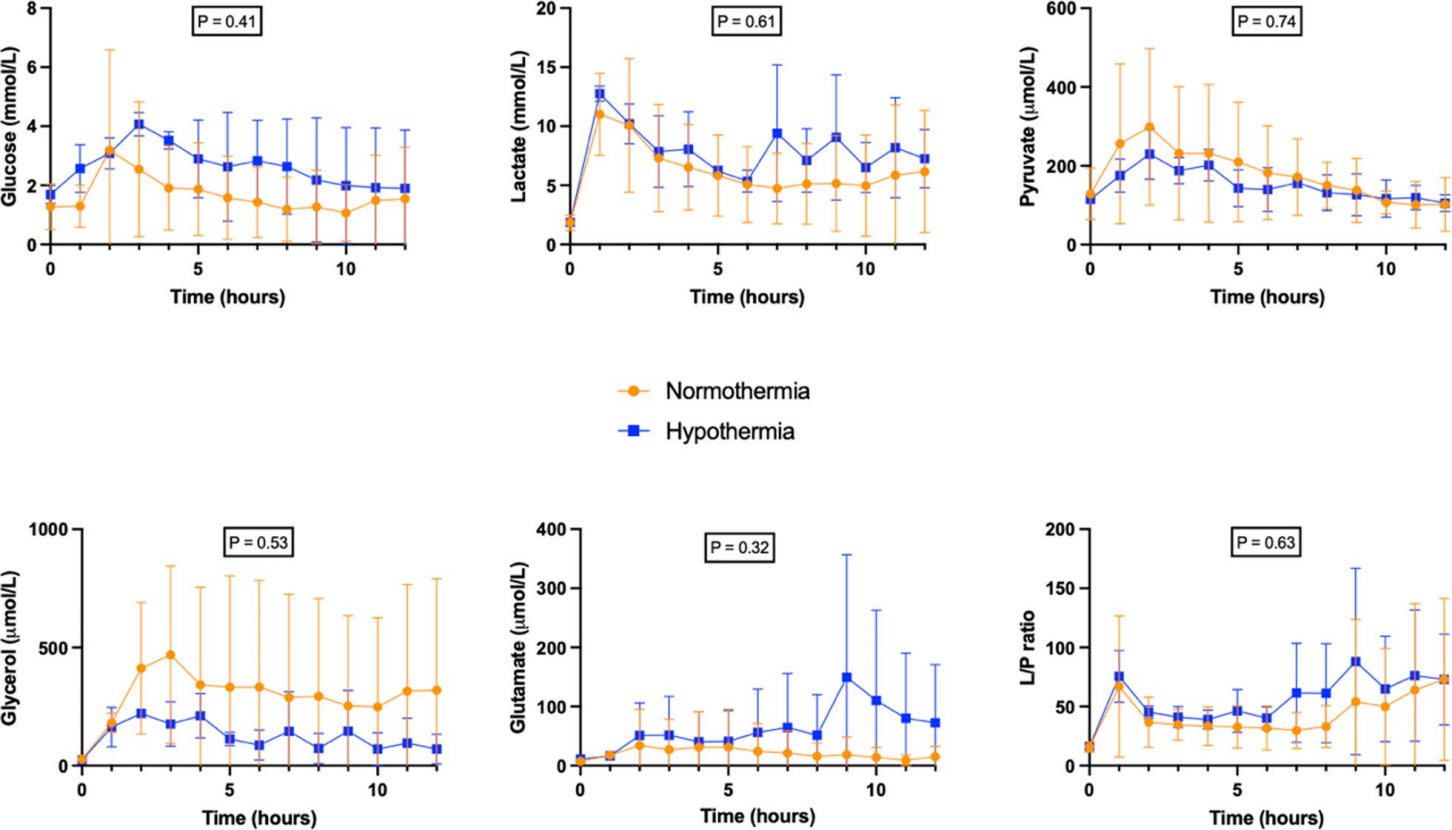
Time-course of cerebral microdialysis parameters throughout the experiment. CMD: cerebral microdialysis; L/P: lactate/pyruvate. *p* *group x time interaction* < 0.05 was considered as statistically significant

After ROSC, no statistically significant difference was observed between the HT group and the NT group regarding glucose (mean difference: − 0.94; 95% CI − 3.69 to + 1.81), lactate (mean difference: – 1.54; 95% CI − 8.87 to + 5.78), pyruvate (mean difference: 27.82; 95% CI − 178.2 to + 233.8), glycerol (mean difference: 170.7; 95% CI − 489.5 to + 830.8), glutamate (mean difference: –40.72; 95% CI − 136.6 to + 55.2) or L/P ratio (mean difference: − 13.3; 95% CI − 81.82 to + 55.22) measured by cerebral microdialysis (Fig. 4).

### Plasma biomarkers

**Fig. 5:**
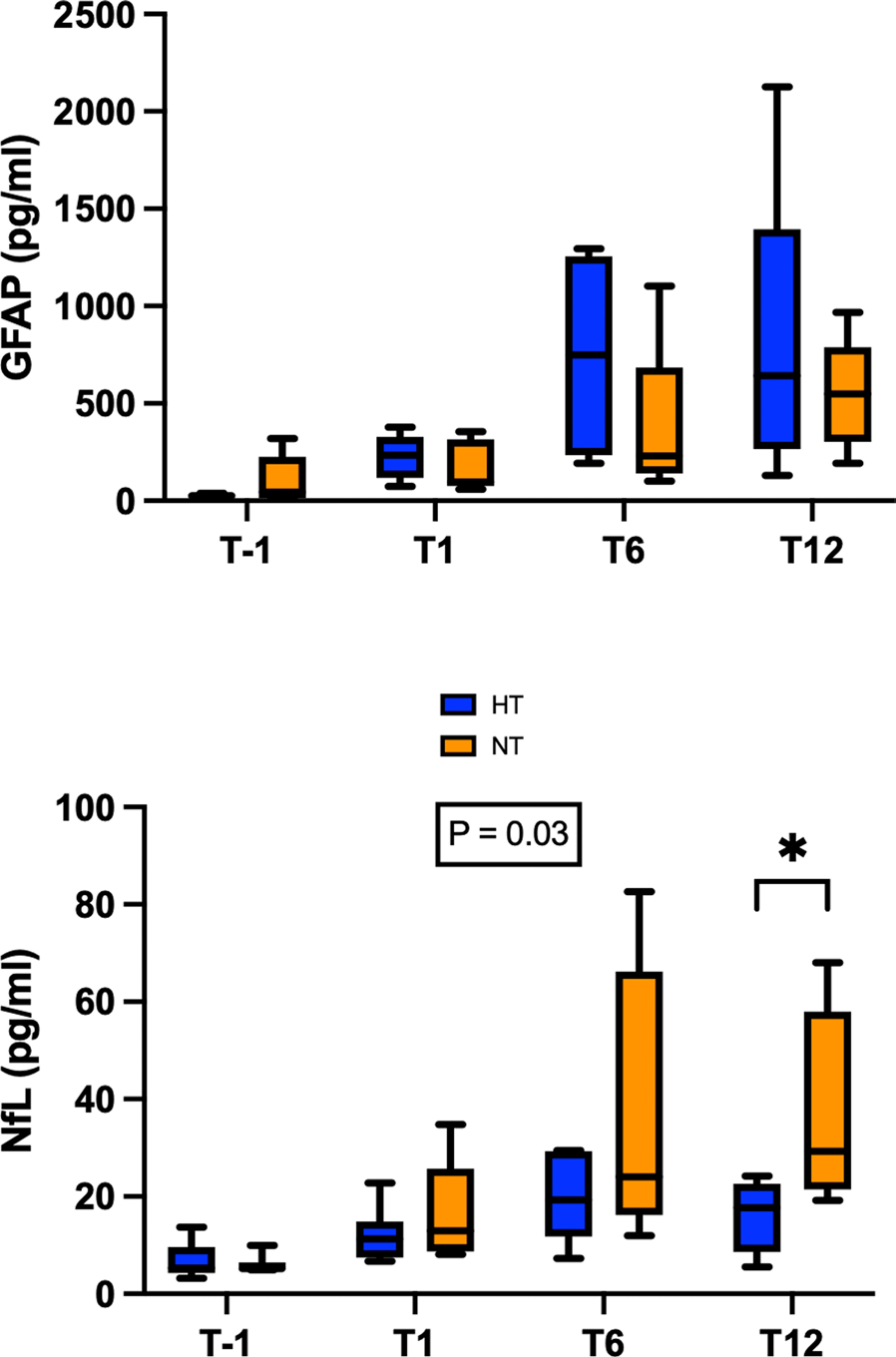
GFAP and NFL levels at baseline (T-1), T1, T6, and T12 post-ROSC. GFAP: glial fibrillary acid protein; NfL: neurofilament light chain protein. *p* *group × time interaction* < 0.05 was considered statistically significant *p < 0.05.

Circulating levels of hemoglobin, white blood cells, platelets, urea nitrogen, creatinine, GOT, GPT, LDH, total and direct bilirubin, troponin I and CPK were similar between the two groups throughout the experiment. Concerning biomarkers of acute brain injury, circulating levels of GFAP did not differ significantly between the two groups at T-1, T1, T6 and T12. Because most baseline NSE values were below the lower limit of detection, NSE results are reported descriptively. At T12, no apparent difference was observed between groups. Notably, the mixed-effects analysis revealed a difference in NfL trajectories between groups, with lower values under hypothermia (*p* *group × time interaction* = 0.03). Using the two-stage linear step-up procedure of Benjamini, Krieger, and Yekutieli, a significant difference was observed at T12 (*p* = 0.04, Fig. 5). For the NfL variable that showed a statistically significant group × time interaction, the corresponding effect sizes and their 95% confidence intervals are presented in the Supplementary Appendix (Table S3).

### Cortical gene expression

**Fig. 6:**
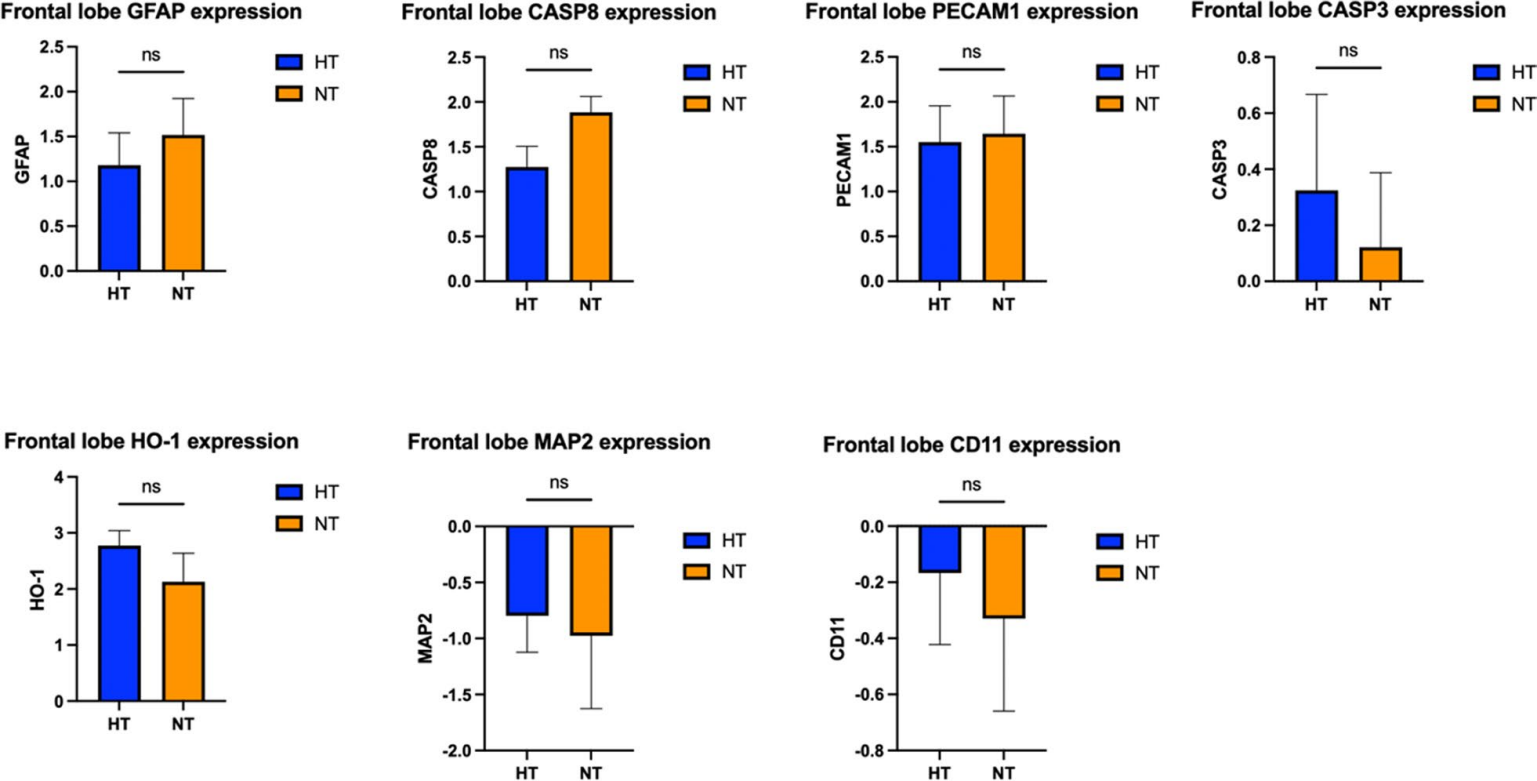
Gene expression in the brain frontal cortex. GFAP: glial fibrillary acid protein; CASP3: caspase 3; CASP8: caspase 8; PECAM1: platelet and endothelial cell adhesion molecule; HO-1 = heme oxygenase 1; MAP2: microtubule-associated protein 2; CD11ß: cluster of differentiation molecule 11ß. *p* < 0.05 was considered as statistically significant

**Fig. 7:**
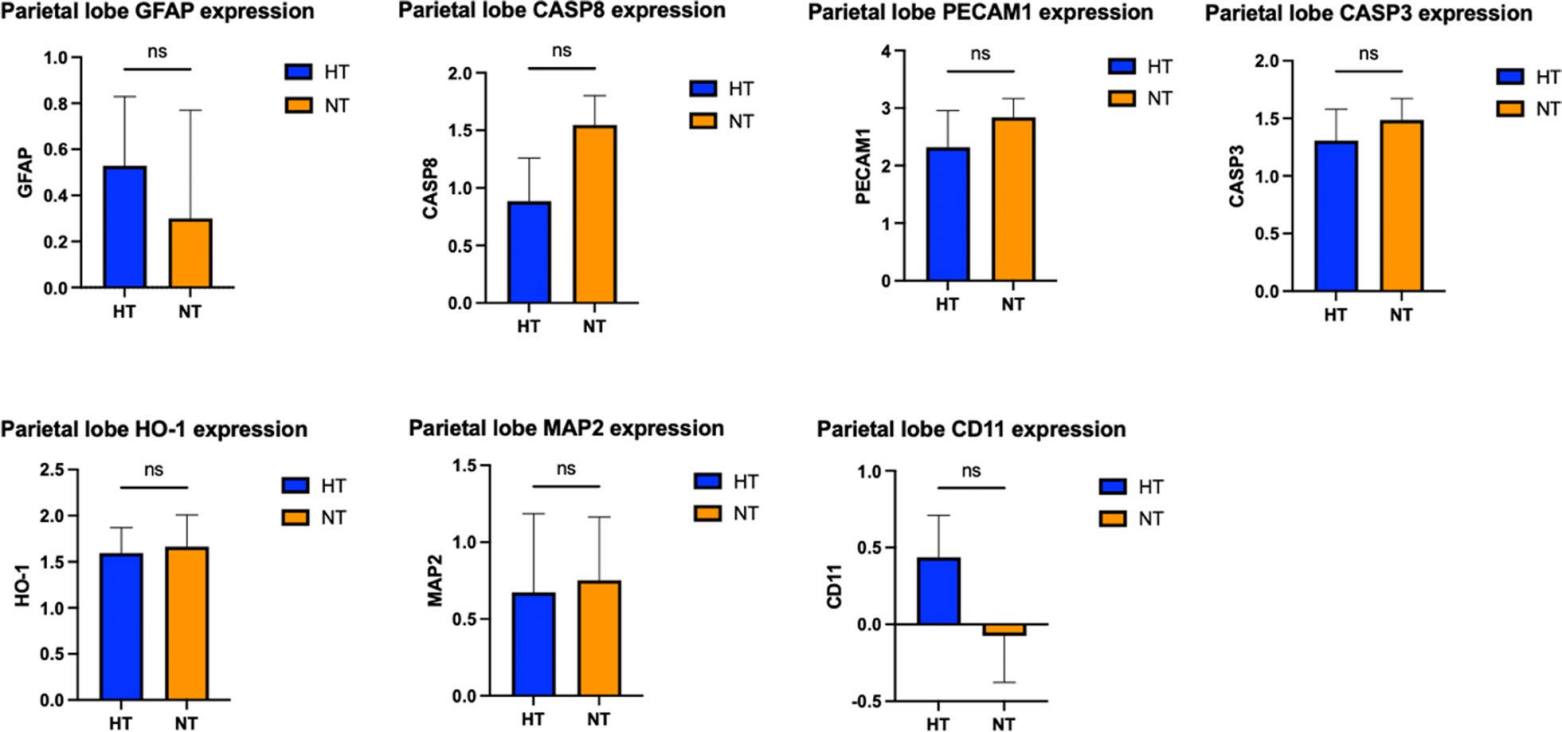
Gene expression in the brain parietal cortex. GFAP: glial fibrillary acid protein; CASP3: caspase 3; CASP8: caspase 8; PECAM1: platelet and endothelial cell adhesion molecule; HO-1 = heme oxygenase 1; MAP2: microtubule-associated protein 2; CD11ß: cluster of differentiation molecule 11ß. *p* < 0.05 was considered as statistically significant

There were no statistically significant differences in the expression of MAP2, GFAP, CD11ß, PECAM1, CASP3, CASP8, and HO-1 between the two groups, whether it is at the level of the frontal lobe (Fig. 6) or for the parietal lobe (Fig. 7).

### Cerebral activity

**Fig. 8:**
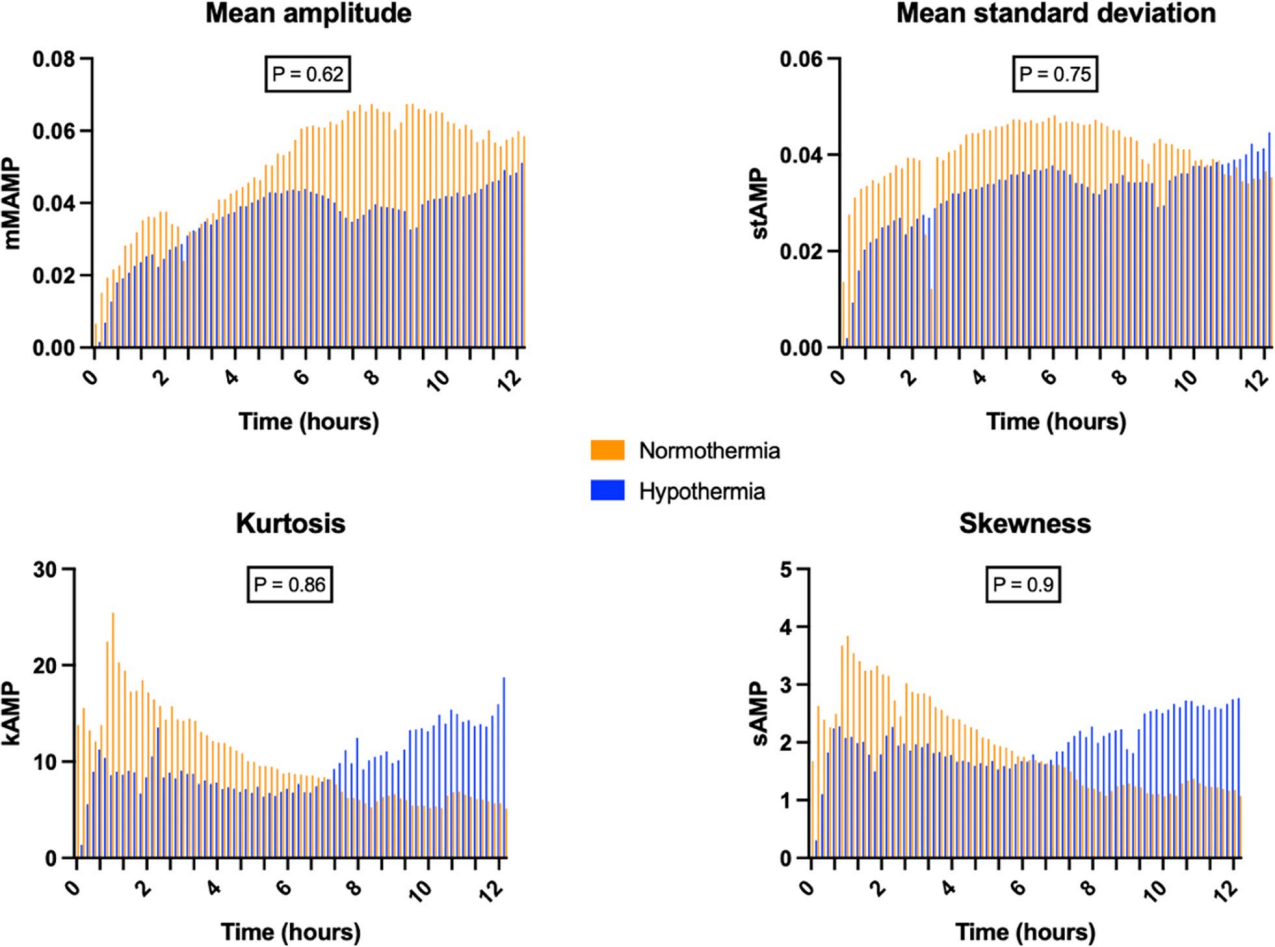
cerebral activity assessed by sEEG. mMAMP: mean amplitude; stamp: mean standard deviation; kAMP: kurtosis; sAMP: skewness. *p* *group x time interaction* < 0.05 was considered as statistically significant

Analysis of continuous EEG revealed lower mean amplitude in the HT group, associated with higher kurtosis and skewness compared to the NT group. However, mean amplitude (mean difference: − 0.014; 95% CI − 0.087 to + 0.058), mean standard deviation (mean difference: − 0.008; 95% CI − 0.07 to + 0.054), kurtosis (mean difference: − 0.51; 95% CI − 7.64 to + 6.61), and skewness (mean difference: 0.051; 95% CI − 1.028 to + 1.13) were not different between the two groups after ROSC (Fig. 8).

## Discussion

Our study showed that, compared to NT, the implementation of HT in a model of refractory cardiac arrest requiring ECPR was not associated with enhanced cerebral perfusion, cerebral extracellular metabolites, or cortical activity. However, NfL levels were lower in the HT group at T12.

HT has been widely studied for its potential neuroprotective effects after CA. By reducing cerebral metabolism and CO₂ production, limiting apoptotic and mitochondrial injury, and attenuating excitotoxicity, inflammation, and free-radical formation, HT may help mitigate ischemia–reperfusion brain damage [7–12]. Despite the theoretical neuroprotective benefits of HT, clinical studies evaluating its impact on neurological outcomes in patients receiving ECPR are not conclusive. An analysis of the existing literature found low-quality evidence pointing toward a possible beneficial effect of HT in the context of ECPR [15]. However, there was significant heterogeneity in HT protocols, particularly in terms of duration, depth, and time to target temperature. Additionally, conducting human studies is challenging due to the need for large sample sizes because of the heterogeneous nature of the included population, characterized by a very high mortality rate, short recruitment windows, and the overall complexity. In this context, animal studies provide a valuable alternative, offering a controlled environment to elaborate therapeutic strategies. Compared to clinical studies, experimental models allow for the standardization of key parameters such as duration of low-flow states, temperature control, and neuromonitoring protocols. Although animal studies have previously explored HT in brain injury models, few have evaluated the effects of HT on brain function using MNM in a refractory cardiac arrest model with ECPR. Regardless of the animal species, six studies [25–30] demonstrated positive effects of HT on brain physiology by either resulting in better neurological performance outcomes (evaluated by neurological scores such as Neurologic Deficit Score or Overall Performance Category), reducing neuronal deaths (assessed by histology and molecular biology), or improving cerebral oxygenation assessed by near-infrared spectroscopy. Nonetheless, the diverse experimental models employed in these ECPR studies limited direct comparisons and external validation of reported findings.

In our study, we aimed to evaluate the impact of systemic HT on cerebral perfusion, metabolism, and cortical activity. Brain perfusion, assessed by ICP measure and PbtO_2_ levels, was similar between the HT group and the NT group. Although there was no significant difference in PbtO_2_ levels between the two groups, PbtO_2_ levels remained superior to 20 mmHg, suggesting that HT had a limited effect on brain tissue oxygenation. Likewise, HT was not associated with ICP level reduction. According to previous clinical and experimental literature, a difference of approximately 5 mmHg in either PbtO₂ or ICP is considered clinically meaningful. In our study, the observed differences between groups in both ICP and PbtO₂ were well below this threshold, indicating no measurable effect of HT on either ICP or PbtO_2_ over the 12-h monitoring period. Importantly, both parameters must be interpreted in the context of ECPR physiology: ECMO-driven PaCO₂ control stabilizes cerebral vasoreactivity and may therefore limit any HT-related changes in cerebral blood flow and tissue oxygenation. This tight regulation of PaCO₂ likely contributed to the absence of differences in PbtO₂ and ICP. As described previously, HT is associated with a reduction in cerebral metabolism and neuroinflammation, which in turn helps reduce cytotoxic and vasogenic edema, leading to a decrease in ICP [7–12]. However, in our model, continuous sedation and prolonged cardiac arrest led to suppressed brain activity in the first hours of the experiment, significantly limiting the metabolism reduction induced by HT. This deep suppression of cerebral metabolism can reduce the physiological window in which HT exerts measurable effects, again making differences in ICP less likely to appear. Additionally, HT is responsible for carbon dioxide reduction and accordingly would further decrease the risk of intracranial pressure [7, 8]. Nevertheless, because ECMO maintained PaCO_2_ at constant levels throughout the experiment, this potential mechanism was blunted, further limiting the impact of HT on brain perfusion. Despite the absence of significant differences in cerebral metabolites assessed by cerebral microdialysis, this finding is consistent with the effects of deep sedation, which reduce cerebral metabolic rate and neuronal activity [31]. Under these conditions, the cerebral metabolic demand is suppressed, potentially masking differences in extracellular metabolite levels. Similarly, the stable PaCO₂ imposed by ECMO may have further homogenized metabolic conditions between groups, contributing to the minimal differences observed in microdialysis measurements.

Although not statistically significant, sEEG analysis revealed a lower mean amplitude in the HT group, associated with higher kurtosis and skewness compared to the NT group. Consistently, the EEG in the HT group appeared globally more attenuated in the early phase, characterized by low-voltage bursts, and evolved more slowly toward a discontinuous suppression–burst pattern, a feature characterized by quasi-periodic alternance of periods of low-voltage brain activity (“suppressions”) and periods of higher voltage activity (“burst”) [32]. In contrast, the NT group demonstrated a faster transition toward a continuous EEG background (Figure S4, Supplementary Appendix). This observation is consistent with the neurophysiological effects of HT on cortical activity. As described by Westover et al. [33], HT leads to a metabolic downregulation of neuronal activity, resulting in reduced burst amplitude and frequency, and prolongation of suppression periods. These changes reflect a state of deep cortical inactivation, with increased intermittency and decreased overall excitability of neurons. Although the differences in kurtosis and skewness did not reach statistical significance, their pattern remains compatible with this interpretation and may reflect a trend toward greater signal irregularity under profound metabolic suppression.

NSE, a cytoplasmic protein expressed in neuronal cell bodies, is the most extensively studied biomarker associated with HIBI. It remains the only biomarker currently recommended for neuro-prognostication following CA [34], where NSE levels typically peak in serum around 72 h post-ROSC [35]. In our study, no significant difference in NSE levels was observed between the two groups at 12 h post-ROSC, likely due to the limited observation period. Similar results were obtained for GFAP, a key protein found mainly in astrocytes, which is consistent with previous findings, as GFAP concentrations are reported to peak between 12 and 48 h following CA [35]. Surprisingly, NfL levels at 12 h post-ROSC were significantly lower in the HT group, despite the biomarker’s delayed peak, typically occurring after 72 h [35]. This finding may reflect an early neuroprotective effect of HT, potentially reducing initial axonal injury and subsequent NfL release. However, given the known early-phase variability of NfL, its delayed kinetics, and the multiple comparisons performed in this study, this isolated difference should be interpreted cautiously and may reflect a type I error.

Our study has several limitations. First, it was conducted in an idealized setting, which may not fully represent the clinical practice. However, we hypothesized that if HT failed to demonstrate a major impact even under optimal conditions, its effectiveness would be even more limited in the clinical setting. Second, the duration of the study was time-limited (12 h), which restricts interpretation to the early reperfusion phase and prevents any assessment of delayed neurological injury or longer-term effects of HT. Third, due to our laboratory logistics constraints, behavioral evaluation could not be performed. Fourth, no histological or immunohistochemical analyses were performed in this study; however, considering the available data, it is unlikely that tissue biopsies assessment would provide significant differences in cerebral injury. Fifth, our study included a relatively small number of animals per group. However, this decision was based on an *interim* statistical analysis, which did not reveal any trend or indication of intergroup differences. Therefore, we considered further inclusion of animals unnecessary and restricted the sample size accordingly. Sixth, because the study was stopped early for futility, the reduced sample size limited the precision of effect estimates, decreased power for secondary endpoints, and may have prevented detection of small or clinically relevant differences. As a result, interpretation of non-significant trends across perfusion, metabolic, and biomarker measures should be made with caution.

## Conclusions

In this study, the use of HT was not associated with a significant improvement in cerebral perfusion or metabolic parameters in a model of refractory cardiac arrest and ECPR. The reduction in NfL levels may suggest a possible neuroprotective effect, which should require further investigation.

## Supplementary Information

Additional file 1.

## Data Availability

The datasets used and/or analyzed during the current study are available from the corresponding author on reasonable request.

## Abbreviations

CA: Cardiac arrest
CT: Cerebral temperature
CPR: Cardiopulmonary resuscitation
ECMO: Extra-corporeal membrane oxygenation
ECPR: Extra-corporeal cardiopulmonary resuscitation
ERC: European Resuscitation Council
ESICM: European Society of Intensive Care Medicine
FiO_2_: Fraction of inspired oxygen
GFAP: Glial fibrillary acidic protein
HIBI: Hypoxic-ischemic brain injury
HT: Hypothermia
ICP: Intracranial pressure
L/P ratio: Lactate/pyruvate ratio
MAP: Mean arterial pressure
MNM: Multimodal neuromonitoring
NfL: Neurofilament light chain
NSE: Neuron-specific enolase
NT: Normothermia
PaCO_2_: Partial pressure of carbon dioxide
PaO_2_: Partial pressure of oxygen
PbtO_2_: Brain oxygen pressure
PPV: Pulse pressure variation
ROSC: Return of spontaneous circulation
sEEG: Stereo-electroencephalography
TTM: Targeted temperature management
VF: Ventricular fibrillation
