# Supplementary material for: Effects of therapeutic hypothermia on brain function in a refractory cardiac arrest model treated with extracorporeal cardiopulmonary resuscitation
Source: Intensive Care Med Exp. 2025 Dec 15;13:127. doi: 10.1186/s40635-025-00841-w (PMC12705496; doi:10.1186/s40635-025-00841-w)

**Supplementary Appendix**

**1. Supplementary Material and Methods**

Power calculation and interim analysis plan

#### **A. Power calculation for the originally planned sample**

The primary endpoint was the **change in brain tissue oxygenation (ΔPbtO₂, mmHg)** over time, analyzed with a **linear mixed-effects model** adjusted for baseline.

Assumptions for the original sample size calculation were:

- Expected mean difference (treatment–control): **Δ = 5 mmHg** (minimal clinically important difference from prior PbtO₂-guided studies).
- Within-subject SD: **σ = 6 mmHg**.
- Correlation between repeated measures: **ρ = 0.6**.
- Number of repeated measurements per animal: **m = 12**.
- Dropout rate : **10%**.
- Two-sided α = 0.05 (O’Brien–Fleming overall), target power = **80%**.

Using these assumptions, sample size of **17 animals per arm** were required to achieve 80% power at α = 0.05.

#### **B. Interim analysis plan**

One interim analysis was pre-specified after inclusion of **n = 6 animals per arm.** The interim analysis was **unblinded**, conducted by the study investigators according to the pre-specified statistical analysis plan. The interim analysis served to evaluate potential **futility.**

- **Alpha spending:** The overall two-sided **α = 0.05** was preserved using an **O’Brien–Fleming–type alpha spending function** (interim boundary p < 0.005; final boundary p < 0.048).
- **Decision rules: Futility:** Stop if **conditional power < 15%** under the assumed treatment effect (Δ = 5 mmHg)

At the interim analysis (n = 6 per arm), **no difference in PbtO₂ was observed**, and the **conditional power was below 15%**, fulfilling the **pre-specified futility criterion**.
The study was therefore **stopped early for futility**, as planned.

**2. Supplementary Tables and Figures**

Figure S1: Time-course of sedation dose (Sevoflurane Minimal Alveolar Concentration), analgesia (morphine infusion rate), respiratory support (minute ventilation) and ECMO sweep gas flow throughout the experiment


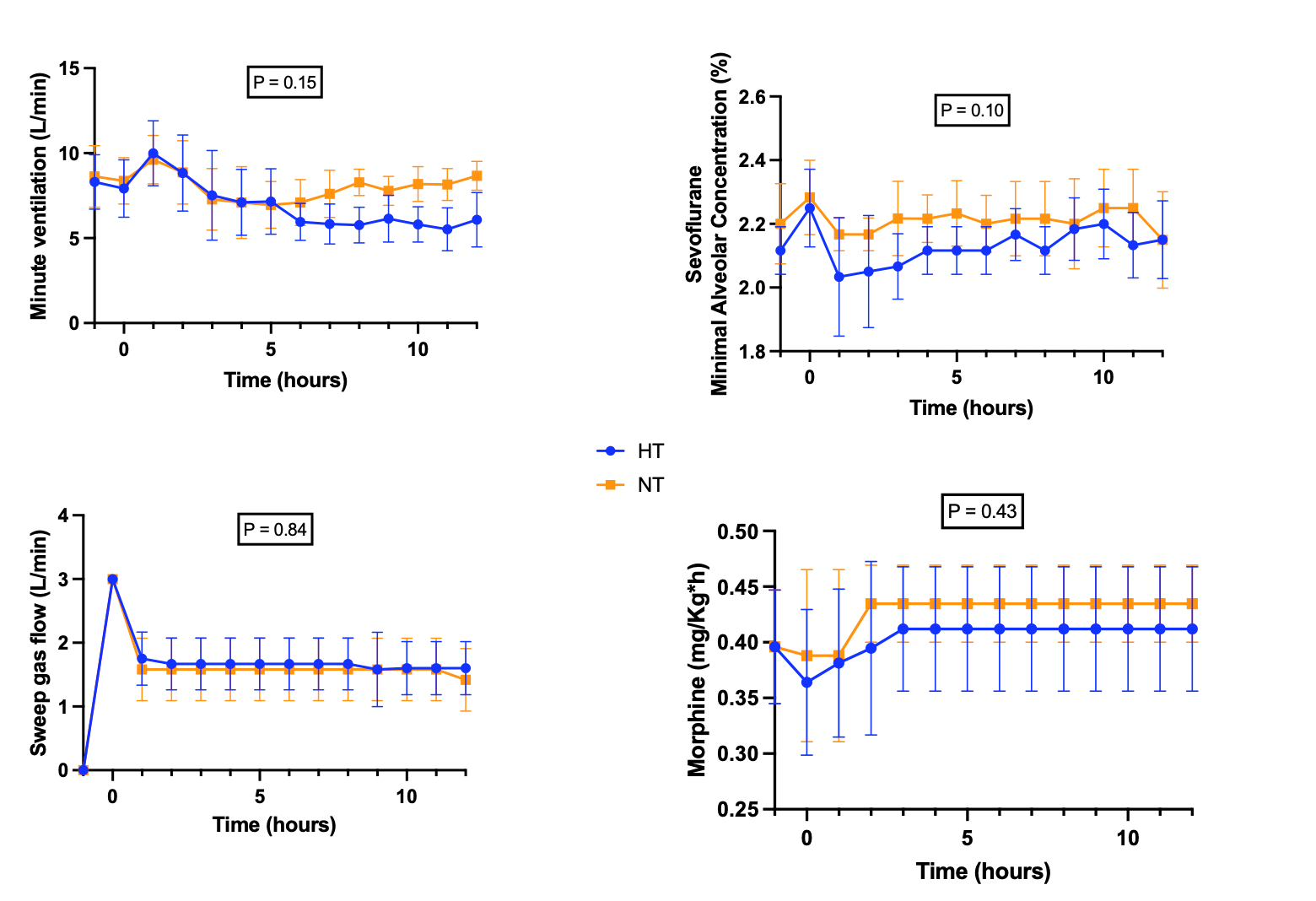


Table S2: Effect Sizes and 95% Confidence Intervals for the Temperature Variable Showing a Significant Group × Time Interaction (p group x time interaction < 0.0001)

| **Mean difference** | **SE of difference** | **95% CIs** |
| --- | --- | --- |
| 0,8429 | 0,2465 | [0,30; 1,39] |
| 1,64 | 0,2465 | [1,10; 2,18] |
| 2,174 | 0,2465 | [1,63; 2,72] |
| 2,664 | 0,2465 | [2,12; 3,21] |
| 3,567 | 0,2558 | [3,00; 4,14] |
| 4,026 | 0,2465 | [3,48; 4,57] |
| 4,183 | 0,2465 | [3,64; 4,73] |
| 4,15 | 0,2558 | [3,58; 4,72] |
| 4,255 | 0,2465 | [3,71; 4,80] |
| 4,363 | 0,2682 | [3,76; 4,97] |
| 4,255 | 0,2465 | [3,71; 4,80] |
| 4,45 | 0,2558 | [3,88; 5,02] |
| 4,323 | 0,2682 | [3,72; 4,93] |
| 4,497 | 0,2682 | [3,89; 5,10] |
| 4,267 | 0,2558 | [3,70; 4,84] |

Table S3: Effect Sizes and 95% Confidence Intervals for the NfL Variable Showing a Significant Group × Time Interaction (p group x time interaction = 0.03)

| **Mean difference** | **SE of difference** | **95% CIs** |
| --- | --- | --- |
| 0,805 | 1,745 | [-3,09; 4,70] |
| -4,725 | 4,817 | [-15,47; 6,02] |
| -16,56 | 12,29 | [-44,34; 11,22] |
| -20,94 | 8,552 | [-40,01; -1,87] |

Figure S4: Representative EEG Trace of an animal from the HT (A) and NT (B) Groups


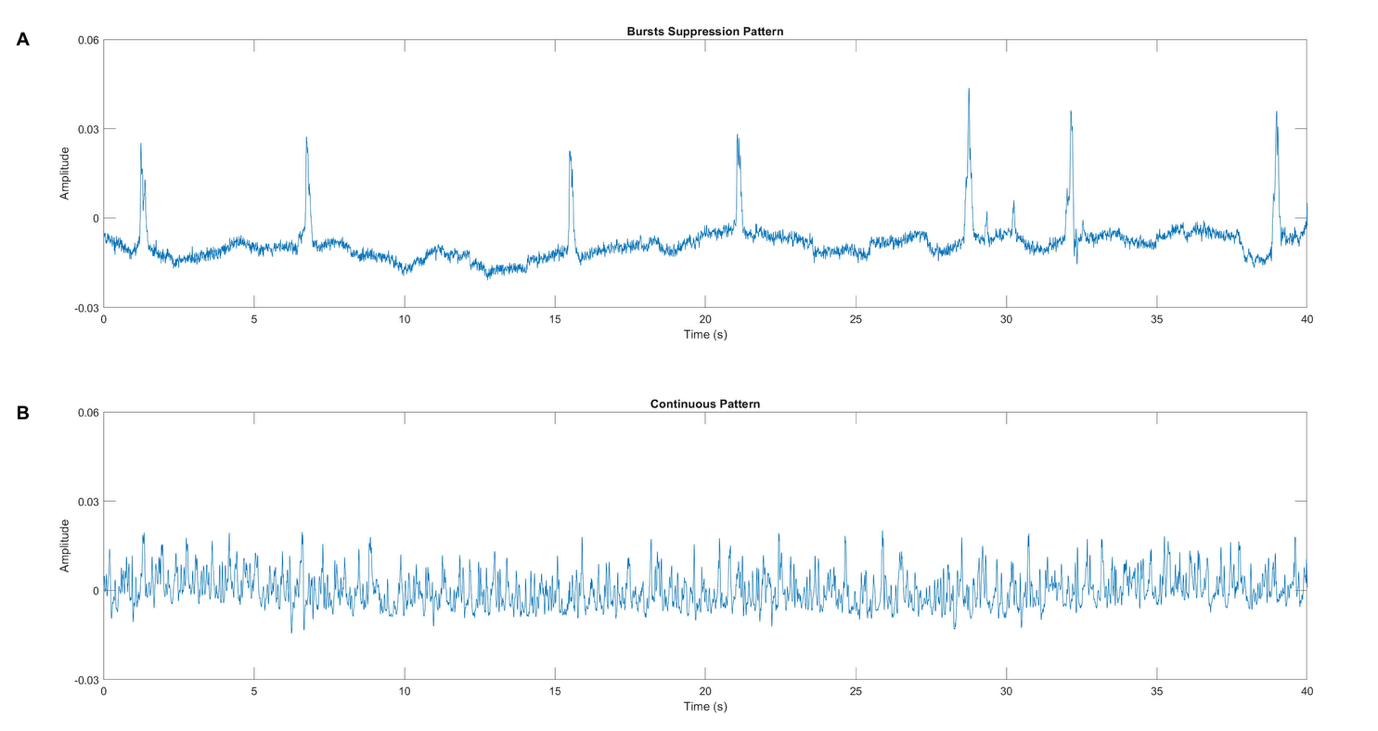

Supplement: Supplementary file 1 — Additional file 1. [file 40635_2025_841_MOESM1_ESM.docx]
